# Prospective study of pain and patient outcomes in the emergency department: a tale of two pain assessment methods

**DOI:** 10.1186/s13049-023-01130-9

**Published:** 2023-10-23

**Authors:** Nai-Wen Ku, Ming-Tai Cheng, Chiat Qiao Liew, Yun Chang Chen, Chih-Wei Sung, Chia-Hsin Ko, Tsung-Chien Lu, Chien-Hua Huang, Chu-Lin Tsai

**Affiliations:** 1 Lawrence S. Bloomberg Faculty of Nursing, University of Toronto, Toronto, ON Canada; 2 Department of Emergency Medicine, National Taiwan University Hospital, 7 Zhongshan S. Rd., Taipei, 100 Taiwan; 3 Department of Emergency Medicine, College of Medicine, National Taiwan University, Taipei, Taiwan; 4 Department of Emergency Medicine, National Taiwan University Hospital Yun-Lin Branch, Hsinchu, Taiwan; 5 Department of Emergency Medicine, National Taiwan University Hospital Hsin-Chu Branch, Hsinchu, Taiwan

**Keywords:** Emergency department, Pain assessment, Triage, Patient outcomes

## Abstract

**Background:**

Accurate pain assessment is essential in the emergency department (ED) triage process. Overestimation of pain intensity, however, can lead to unnecessary overtriage. The study aimed to investigate the influence of pain on patient outcomes and how pain intensity modulates the triage’s predictive capabilities on these outcomes.

**Methods:**

A prospective observational cohort study was conducted at a tertiary care hospital, enrolling adult patients in the triage station. The entire triage process was captured on video. Two pain assessment methods were employed: (1) Self-reported pain score in the Taiwan Triage and Acuity Scale, referred to as the system-based method; (2) Five physicians independently assigned triage levels and assessed pain scores from video footage, termed the physician-based method. The primary outcome was hospitalization, and secondary outcomes included ED length of stay (EDLOS) and ED charges.

**Results:**

Of the 656 patients evaluated, the median self-reported pain score was 4 (interquartile range, 0–7), while the median physician-rated pain score was 1.5 (interquartile range, 0–3). Increased self-reported pain severity was not associated with prolonged EDLOS and increased ED charges, but a positive association was identified with physician-rated pain scores. Using the system-based method, the predictive efficacy of triage scales was lower in the pain groups than in the pain-free group (area under the receiver operating curve, [AUROC]: 0.615 vs. 0.637). However, with the physician-based method, triage scales were more effective in predicting hospitalization among patients with pain than those without (AUROC: 0.650 vs. 0.636).

**Conclusions:**

Self-reported pain seemed to diminish the predictive accuracy of triage for hospitalization. In contrast, physician-rated pain scores were positively associated with longer EDLOS, increased ED charges, and enhanced triage predictive capability for hospitalization. Pain, therefore, appears to modulate the relationship between triage and patient outcomes, highlighting the need for careful pain evaluation in the ED.

**Supplementary Information:**

The online version contains supplementary material available at 10.1186/s13049-023-01130-9.

## Introduction

Pain assessment is a fundamental component of the triage process in the emergency department (ED). Pain assessment is also frequently used, as approximately 60% of ED visits result from pain-related problems [1–3]. Recognizing the significance of this, the American Pain Society (APS) designated pain as the “fifth vital sign” in the 1990s [4]. Instruments such as the numeric rating scales (NRS) and visual analog scales (VAS) are commonly used for self-reported pain evaluation in EDs [5, 6]. Yet, multiple studies suggest that subjective numeric pain scores may not necessarily enhance pain management or improve outcomes when designated as a fifth vital sign [4, 7, 8].

A fundamental criterion for triage systems is the accurate identification of patients with critical, life-threatening, or significant injuries [9, 10]. In Taiwan, the Taiwan Triage and Acuity Scale (TTAS) has been widely used for more than a decade. It was derived from the Canadian Triage and Acuity Scale (CTAS) and has been validated against parameters such as hospital admission, ED length of stay, and resource utilization [9, 11]. Importantly, within the TTAS, a self-reported pain score acts as a vital modifier. Should patients report elevated pain scores, the TTAS computerized system automatically assigns them to higher triage categories [12]. Existing literature suggests that integrating inherently subjective pain assessments into triage may lead to potential overestimation or misrepresentation of patient triage categories [2, 13]. Consequently, patients expressing heightened pain could be prioritized above those who are ill but do not express severe pain [2]. The exact influence of pain on the correlation between triage and patient outcomes remains unclear, underscoring the need for continued investigation. For this purpose, we utilized two distinct pain assessment methodologies: a system-based approach that relies on subjective evaluation, and a physician-based approach that offers a more objective assessment.

The current study aimed to compare the distributions of pain intensity and its relationship with outcomes (namely, hospital admission, ED length of stay, and ED charges) across both the system-based and physician-based approaches. Additionally, we explored the modulation effect of pain intensity on the association between triage and patient outcomes. We hypothesized that, with the system-based method, pain would attenuate the predictive capability of triage in relation to patient outcomes.

## Methods

### Study design, setting, and population

The study design is detailed in a prior publication [14]. Briefly, a prospective observational cohort study was undertaken at a medical center hospital between May 2020 and June 2021. All patients aged 20 years or older (the legal majority age in Taiwan) presenting to the ED were screened for their eligibility and were enrolled by trained research staff following a standardized protocol. Exclusion criteria included patients in need of immediate cardiopulmonary resuscitation, those under isolation for potential infectious diseases, and individuals with communication barriers. The detailed inclusion and exclusion criteria were outlined in Additional file 1: Table S1. Ethical approval was granted by the National Taiwan University Hospital Institutional Review Board, with informed consent secured from all participants. The presentation of results adhered to the Strengthening the Reporting of Observational Studies in Epidemiology (STROBE) guidelines [15].

### System-based method

The TTAS is employed to identify and prioritize patients requiring urgent attention based on the severity of their medical conditions. This system categorizes patients into five distinct levels: Level 1 (resuscitation), Level 2 (emergent), Level 3 (urgent), Level 4 (less urgent), and Level 5 (non-urgent) [9]. The allocation to a triage level in TTAS relies primarily on the patient’s chief complaints. Additionally, vital signs—such as body temperature, heart rate, respiratory rate, systolic and diastolic blood pressure, and oxygen saturation—pain intensity, and the mechanism of injury are crucial determinants. Additional data, including consciousness level, pre-existing comorbidities, demographic information, and the mode of arrival are also taken into account. This comprehensive assessment is conducted by senior ED triage nurses proficient in computerized systems. Following this evaluation, nurses choose the relevant decision pathway, and the triage level is subsequently displayed.

The TTAS triage algorithm utilizes an NRS to systematically evaluate self-reported pain intensity, ranging from 0 to 10. A score of 0 signifies no pain, while a score of 10 denotes maximal pain intensity [5]. Within the TTAS framework, any reported acute central pain with a score exceeding eight triggers the computerized system to elevate the triage level by one, with a ceiling of Level 2. For the purposes of this study, the NRS was stratified into four categories: pain-free (0), mild (1–3), moderate (4–7), and severe pain (8–10) (9, 10). Moreover, we explored the influence of using the pain modifier during the triage, where the triage nurses could choose to classify patients with pain-related complaints primarily based on pain (i.e., using pain as a primary decision path). Not all patients with pain-related chief complaints will undergo this path, especially those who have multiple complaints. Overall, the triage approach that relies on the self-reported pain score, is referred to as the system-based method.

### Physician-based method and video review

The entire triage procedure was documented via video recording. A research assistant verified the quality of these recordings. Subsequently, five physicians were provided access to the electronic triage records, with both system-determined triage levels and patient-reported pain scores obscured. By examining the full videos, the physicians derived a conclusive triage level based on perceived urgency and pain score based on objective indicators such as facial expressions, vocal cues, primary complaints, and vital signs. This technique is denoted as the physician-based method, which combines physician-determined triage and physician-rated pain scores.

For this study, the first five videos served as pilot data and were evaluated by all reviewers. The consistency in perceived triage levels and pain scores among reviewers was determined using the intraclass correlation coefficient (ICC). The ICC values for this pilot dataset stood at 0.59 and 0.53, reflecting moderate consensus. Following this pilot phase, the reviewers independently evaluated the subsequent video recordings.

### Outcomes

The primary outcome assessed was subsequent hospitalization following the ED visit. Secondary outcomes included the ED length of stay (EDLOS) and the associated ED charges. EDLOS is defined as the interval from ED triage to either hospital admission or ED release. All ED charges, denominated in New Taiwan Dollars (NT$), encompassed registration charges, physician fees, medication charges, and out-of-pocket charges.

### Statistical analysis

Data were input and analyzed utilizing IBM SPSS Statistics (version 26.0, IBM, Armonk, NY, USA). Continuous variables were described using means and standard deviations (SDs), while categorical variables were summarized with counts and percentages. Medians and interquartile ranges (IQRs) were reported for EDLOS and emergency department charges, as their distributions were right-skewed. A *p* for trend test was employed to examine the relationship between the two pain assessment methods and their outcomes. To determine the predictive ability for hospitalization, the area under the receiver operating characteristic (AUROC) curve was utilized to assess the discriminatory performance of triage levels in the context of both system-based and physician-based methods. A subgroup analysis was conducted comparing the pain-free group to the pain modifier group within the system-based method. All statistical tests were two-sided, with a significance level set at 0.05.

## Results

**Fig. 1:**
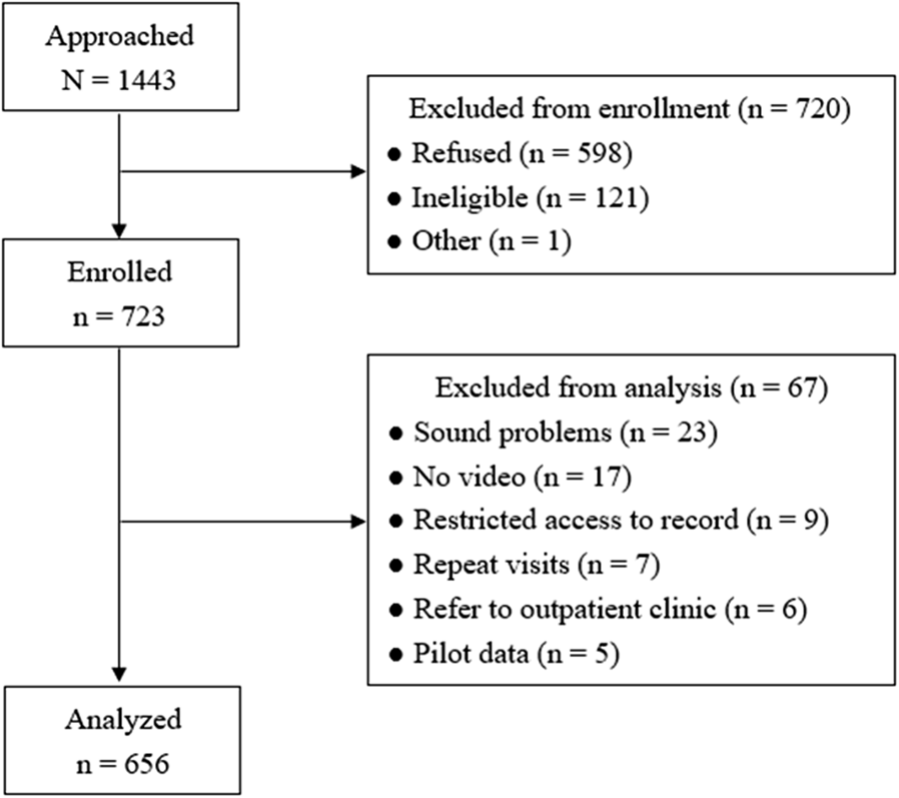
Flow diagram of the patient enrollment process

The patient selection process is illustrated in Fig. 1. Initially, 1,443 patients were considered; however, 598 declined participation, while 122 were found ineligible for various reasons. This led to the enrollment of 723 patients. Subsequently, 67 patients were removed due to audiovisual issues, repeated visits, or medical record constraints. As a result, 656 patients constituted the final cohort for analysis.

The clinical characteristics of the patients are summarized in Table 1. The mean age was 52.3 years, with 330 (50.3%) being male. Using the system-based method, most patients were assigned to Level 3, followed by Level 2, and Level 4. The median self-reported pain score was 4 (IQR, 0–7). Most (44.4%) of patients reported moderate pain, followed by no pain (43.6%), severe pain (9.6%), and mild pain (2.4%). By contrast, with the physician-based method, most patients were categorized at Level 4, followed by Level 3, and Level 5. The median physician-rated pain score was only 1.5 (IQR, 0–3). Physicians rated most patients (41.6%) as mild pain, followed by no pain (26.9%) and moderate pain (19.5%). The triage process took approximately 2–3 min. Approximately 16% of the patients were admitted for both medical and surgical reasons.

**Table 1:** Baseline clinical characteristics of emergency department patients

Variable	(n = 656)
Demographics	
Age, mean (SD), year	52.3 (18.6)
Male sex, n (%)	330 (50.3)
System-based method	
System triage level, median (IQR)	3 (3–3)
System triage level, n (%)	
1	3 (0.5)
2	84 (12.8)
3	491 (74.9)
4	66 (10.1)
5	12 (1.8)
Self-reported pain score, median (IQR)	4 (0–7)
Self-reported pain intensity, n (%)	
Pain-free	286 (43.6)
Mild (1–3)	16 (2.4)
Moderate (4–7)	291 (44.4)
Severe (8–10)	63 (9.6)
Physicians-based method	
Physician-based triage level, median (IQR)	4 (3–4)
Physician-based triage level, n (%)	
1	2 (0.3)
2	43 (6.6)
3	171 (26.1)
4	280 (42.7)
5	160 (24.4)
Physician-rated pain score, median (IQR)	1.5 (0–3)
Physician-rated pain intensity, n (%)	
Pain-free	242 (26.9)
Mild (1–3)	273 (41.6)
Moderate (4–7)	128 (19.5)
Severe (8–10)	13 (2.0)
Triage duration, median (IQR), minutes: seconds	2:25 (1:53–3:10)
Hospital admission (n = 108), n (%)	
Intra-abdominal infection	14 (13.0)
Cerebrovascular accident	8 (7.4)
Urinary tract infection	7 (6.5)
Pneumonia	6 (5.6)
Cellulitis	6 (5.6)
Fractures	6 (5.6)

SD = standard deviation; IQR = interquartile range

**Fig. 2:**
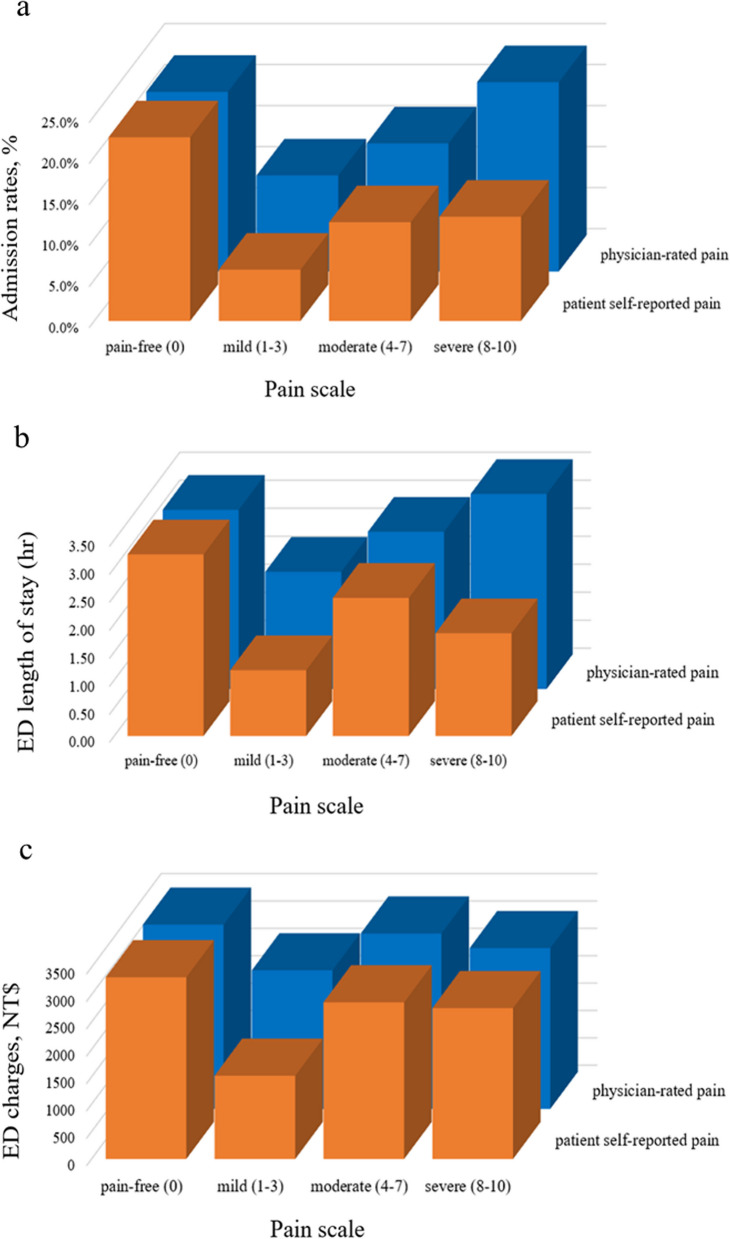
Hospital admission, emergency department length of stay, and emergency department charges by pain group in each pain assessment method

The comparison between two pain assessment methods and their subsequent impact on patient outcomes, specifically hospital admission, EDLOS, and ED charges, are depicted in Fig. 2a–c. Figure 2a presents the hospital admission rates derived from both self-reported and physician-rated pain scores. Patients who self-reported moderate and severe pain demonstrated similar admission rates (12.0% and 12.7%, respectively). A mere 6.3% of patients self-reporting mild pain were hospitalized. Overall, self-reported pain scores displayed no significant correlation with hospital admission (*P* trend = 0.611). In contrast, while there was no significant correlation between physician-rated pain scores and hospitalization (*P* trend = 0.141), there was a noticeable upward trend in admission rates with increasing pain severity within the physician-rated group: 11.7%, 15.6%, and 23.1% for mild, moderate, and severe pain, respectively. Notably, both assessment methods yielded comparable admission rates for the pain-free group (22.4 vs. 21.9%).

Figure 2b delineates the median EDLOS according to self-reported and physician-rated pain categories. There was no significant association between self-reported pain intensity and EDLOS (*P* trend = 0.514). For the self-reported categories, patients with moderate pain had an average EDLOS of 2.5 h, followed by those with severe pain (1.8 h) and mild pain (1.2 h). In stark contrast, the EDLOS demonstrated an upward trajectory for the physician-rated group with increasing pain intensity, registering 2.1 h for mild pain, 2.8 h for moderate pain, and 3.5 h for severe pain. A significant association was observed between increased physician-rated pain scores and extended EDLOS (*P* trend < 0.0001). Notably, the median EDLOS for the pain-free group was consistent across both assessment methods, standing at 3.2 h.

Figure 2c depicts the median ED charges based on self-reported and physician-rated pain scores. Self-reported pain scores showed no significant association with ED charges (P trend = 0.853). Charges for the moderate and severe self-reported pain groups were 2861 NT$ and 2755 NT$, respectively, while the mild pain group had the lowest charges at 1521 NT$. Conversely, physician-rated pain scores increased with rising ED charges (*P* trend = 0.002). Among the physician-rated categories, the moderate pain group incurred greater ED charges (3202 NT$) than the severe (2936 NT$) and mild pain groups (2530 NT$). Interestingly, both assessment methods produced comparable ED charges for the pain-free group (3318 NT$ vs. 3364 NT$).

**Fig. 3:**
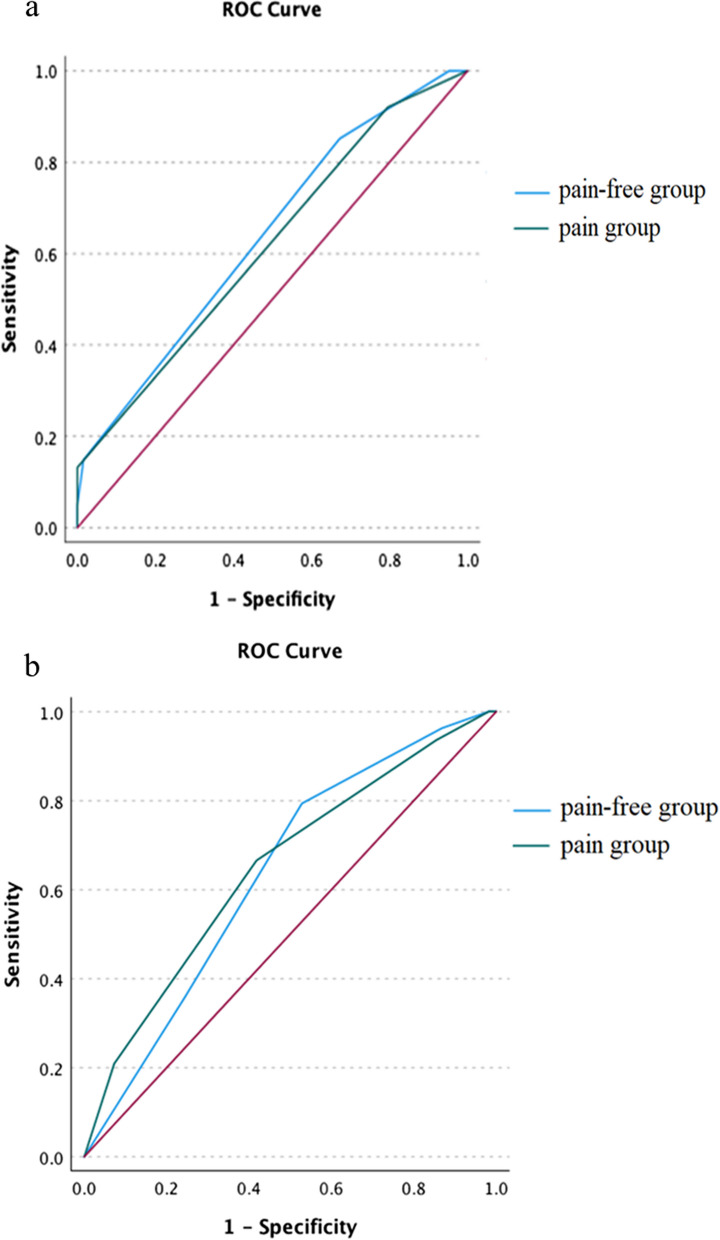
The receiver operating characteristic curves for predicting hospital admission by pain group in each pain assessment method

Table 2 presents a comparison of AUROCs between the pain-free and pain groups for both system-based and physician-based methods. For the system-based method, the AUROCs for the pain-free group and pain group were 0.637 (95% CI 0.561, 0.713) and 0.615 (95% CI 0.530, 0.700), respectively. While the difference was not statistically significant (*P* = 0.705), the AUROC for the pain-free group was marginally higher, as depicted in Fig. 3a.

**Table 2:** Areas under the receiver operating curve and 95% confidence intervals of the logistic regression models for hospital admission by pain groups

Outcome	AUC of the system-based method	
Hospital admission	Pain-free (n = 286)	Pain (n = 370)
	0.637 (0.561, 0.713)	0.615 (0.530, 0.700)

Outcome	AUC of the physician-based method	
Hospital admission	Pain-free (n = 242)	Pain (n = 414)
	0.636 (0.545, 0.726)	0.650 (0.573, 0.726)

AUC: Area under the receiver operating curve

In the physician-based method, the AUROCs for the pain-free and pain groups were 0.636 (95% CI 0.545, 0.726) and 0.650 (95% CI 0.573, 0.726), respectively. While the difference was not statistically significant (*P* = 0.818), the pain group showed a slightly elevated AUROC, as illustrated in Fig. 3b.

In a subgroup analysis, the pain modifier subgroup and the pain-free group had AUROCs of 0.606 (95% CI 0.506, 0.705) and 0.637 (95% CI 0.561, 0.713), respectively, as shown in Additional file 2: Table S2, Additional file 3: Fig. S1. The pain modifier subgroup exhibited a reduced AUROC compared to the pain-free group, though the difference was not clinically significant (*P* = 0.623).

## Discussion

In this prospective study, we employed videotaped recordings to investigate the association between pain and patient outcomes using two distinct approaches: system-based and physician-based methods. Our findings elucidated three pivotal insights: (1) Pain intensity exhibited no correlation with extended EDLOS and augmented ED charges with the self-reported pain scores; however, a positive association was evident with the physician-rated pain scores. (2) Incorporating the subjective pain assessment (e.g., NRS) into the ED triage might diminish the triage’s predictive capacity regarding hospitalization; (3) in contrast, incorporating the objective pain assessment (e.g., physician-rated method) into the ED triage might strengthen the triage’s predictive capacity regarding hospitalization.

Whether the intensity of pain related to outcomes seemed to depend on the method of pain assessment. Our findings revealed no significant correlation between pain intensity and both EDLOS and ED charges with the self-reported pain scores. A positive correlation, however, was found with the physician-rated scores. Previous research suggested that patients based their pain scores on their emotional and sensory experiences at the moment [2, 13, 16]; they would not and probably could not foresee their subsequent health outcomes. Although the NRS is a quick and straightforward pain assessment tool, patients may struggle to provide accurate responses within the restricted timeframe of ED triage [6, 8, 17]. In contrast, this correlation with the physician assessment may arise from healthcare providers’ perception that the primary purpose of pain scoring is to raise awareness and trigger intervention [18], leading clinicians to focus on identifying patients at risk of critical conditions rather than on patients’ subjective pain experiences [2, 6]. For example, while not statistically significant, there was a noticeable trend of increasing admission rates with pain intensity in the physician-rated groups, as illustrated in Fig. 2a. Notably, both physician-rated and self-reported pain-free groups yielded comparable results across all three outcomes. This aligns with prior research indicating that pain, due to its subjective nature potentially influenced by many factors, such as age, gender, and education, may not be a dependable predictor of outcomes [1, 19, 20]. Hence, pain assessment in the ED should be approached with discernment, given its possible influence on triage level and timeliness of treatment.

Incorporating subjective pain assessments into ED triage may compromise the triage’s predictive ability regarding patient outcomes. Our results showed that the pain-free group exhibited a superior AUROC compared to the pain group when using the system-based approach. Furthermore, the subgroup analysis of pain modifiers displayed the lowest AUROC. This suggests that incorporating self-reported pain may disrupt the correlation between triage and hospitalization, corroborating earlier studies [2, 13, 16]. Potential reasons might encompass inadequate pain evaluation leading to mis-triage and overestimation of patient severity [2, 13]. Davis et al. posited that excluding pain assessment from the CTAS would not diminish the triage’s capacity to forecast mortality and hospitalization. Moreover, upon examining pain subgroups, such as abdominal pain, cardiac-related chest pain, and headache, while omitting numeric pain scores, no significant discrepancies in triage ability to predict outcomes were noted [2]. This underscores the possibility that pain may not serve as an essential modifier in the system-based approach, given its potential to undermine the triage’s prognostic capabilities.

Conversely, the AUROC for the pain group using the physician-based method was superior to that of the pain-free group. Previous studies have suggested that healthcare professionals may correlate pain intensity with the severity of the disease or its ramifications on a patient’s daily activities [4, 18]. In this study, physicians noted that the pain-free cohort evaluated by the system-based method might have exhibited unreported pain, as indicated by nonverbal cues, including facial expressions, functional activity, and communication patterns [21]. Furthermore, upon arriving at the ED, patients might prioritize more pronounced symptoms, such as vertigo or fever, potentially overlooking subtle pain or emotional distress [22–24]. Moreover, patients with severe illness, cognitive impairment, or delirium may struggle to provide accurate self-reported pain scores even when they have pain-related complaints [2, 25, 26]. Collectively, these findings suggest that physician-rated pain scores may correctly reclassify self-reported pain intensity and improve the predictive accuracy of triage for hospital admission.

This study has certain limitations that should be considered. Firstly, being a prospective cohort study, it included participants from only one medical center, constraining the generalizability of the findings to other institutions. Moreover, the outcomes of the present study cannot be extrapolated to pediatric populations as the inclusion criteria were exclusively focused on adults.

## Conclusions

In this study, self-reported pain did not exhibit a significant relationship with patient outcomes and appeared to diminish the predictive efficacy of triage for hospitalization. In contrast, physician-rated pain demonstrated a positive correlation with extended EDLOS and increased ED charges. Moreover, it strengthened the predictive ability of triage for hospitalization. Further research is warranted to develop objective pain assessment tools within the ED setting. Accurate pain assessment could enhance triage accuracy, alleviate the workload on triage nurses, and potentially improve patient outcomes.

### Supplementary Information

**Additional file 1. Supplementary Table 1. **Inclusion and exclusion criteria for this study.

**Additional file 2. Supplementary Table 2.** Areas under the receiver operating curve and 95% confidence intervals of the logistic regression models for hospital admission within the system-based method.

**Additional file 3. Supplementary Figure 1.** The receiver operating characteristic curves of the logistic regression models for hospital admission within the system-based method, subgroup analysis.

## Data Availability

The datasets used and/or analyzed during the current study are available from the corresponding author on reasonable request.

## Abbreviations

ED: Emergency department
EDLOS: ED length of stay
APS: American pain society
NRS: Numeric rating scales
VAS: Visual analog scales
TTSA: Taiwan triage and acuity scale
CTAS: Canadian triage and acuity scale
