# Supplementary material for: Prospective study of pain and patient outcomes in the emergency department: a tale of two pain assessment methods
Source: Scand J Trauma Resusc Emerg Med. 2023 Oct 23;31:56. doi: 10.1186/s13049-023-01130-9 (PMC10594810; doi:10.1186/s13049-023-01130-9)
Supplement: Supplementary file 1 — Additional file 1. Supplementary Table 1. Inclusion and exclusion criteria for this study. [file 13049_2023_1130_MOESM1_ESM.docx]

**Supplementary Table 1.** Inclusion and exclusion criteria for this study.

| Inclusion criteria | Exclusion criteria |
| --- | --- |
| - All patients aged 20 years or older presenting to the ED | - In need of immediate cardiopulmonary resuscitation - Require isolation for potential infectious diseases (e.g., COVID-19 and tuberculosis) - With communication barriers (e.g., coma or intoxication and lack of a caregiver who could act as a proxy to give informed consent) - Refused participation - Language barriers and without an interpreter |
