# Supplementary material for: Prospective study of pain and patient outcomes in the emergency department: a tale of two pain assessment methods
Source: Scand J Trauma Resusc Emerg Med. 2023 Oct 23;31:56. doi: 10.1186/s13049-023-01130-9 (PMC10594810; doi:10.1186/s13049-023-01130-9)
Supplement: Supplementary file 2 — Additional file 2. Supplementary Table 2. Areas under the receiver operating curve and 95% confidence intervals of the logistic regression models for hospital admission within the system-based method. [file 13049_2023_1130_MOESM2_ESM.docx]

**Online Supplementary Table 2**. Areas under the receiver operating curve and 95% confidence intervals of the logistic regression models for hospital admission within the system-based method.

| **AUC of the system-based method** | |  |
| --- | --- | --- |
| Main analysis | |  |
| Pain-free  (n=286) | Pain  (n=370) | |
| 0.637 (0.561, 0.713) | 0.615 (0.530, 0.700) | |
| Subgroup analysis |  | |
| Pain-free  (n=286) | Subgroup with a pain modifier  (n=297) | |
| 0.637 (0.561, 0.713) | 0.606 (0.506, 0.705) | |
