# Supplementary figures and images for: Prospective study of pain and patient outcomes in the emergency department: a tale of two pain assessment methods
Source: Scand J Trauma Resusc Emerg Med. 2023 Oct 23;31:56. doi: 10.1186/s13049-023-01130-9 (PMC10594810; doi:10.1186/s13049-023-01130-9)

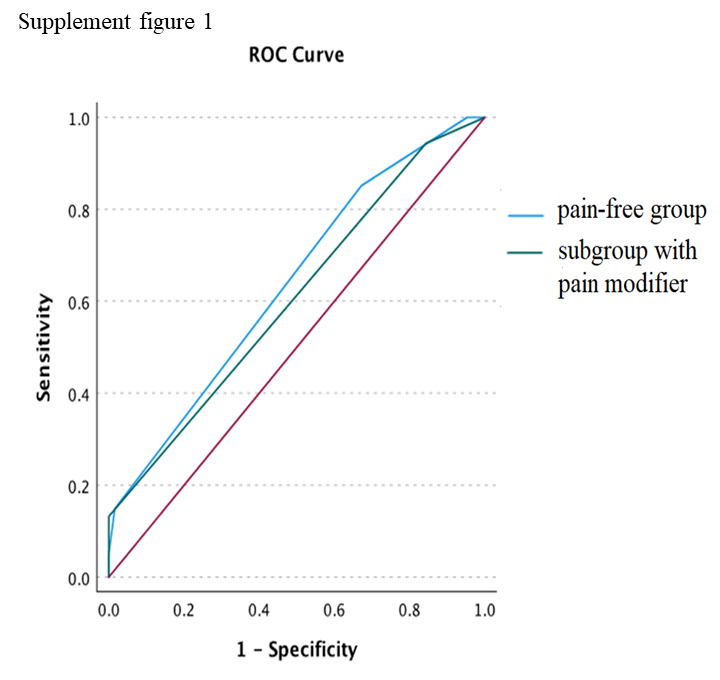

Supplement: Supplementary file 3 — Additional file 3. Supplementary Figure 1. The receiver operating characteristic curves of the logistic regression models for hospital admission within the system-based method, subgroup analysis. [file 13049_2023_1130_MOESM3_ESM.tiff]
